# Concepts and Analyses in the CT Scanning of Root Systems and Leaf Canopies: A Timely Summary

**DOI:** 10.3389/fpls.2015.01111

**Published:** 2015-12-24

**Authors:** Jonathan A. Lafond, Liwen Han, Pierre Dutilleul

**Affiliations:** ^1^Département des Sols et de Génie Agroalimentaire, Université Laval, QuébecQC, Canada; ^2^Environmetrics Laboratory, Department of Plant Science, McGill University, MontréalQC, Canada

**Keywords:** computed tomography scanning, root systems and leaf canopies, scale of observation vs. scale of resolution, CT image processing and CT number analysis, structural complexity and fractal geometry, repeated plant CT scanning and statistical aspects, multidisciplinary applications

## Abstract

Non-medical applications of computed tomography (CT) scanning have flourished in recent years, including in Plant Science. This Perspective article on CT scanning of root systems and leaf canopies is intended to be of interest to three categories of readers: those who have not yet tried plant CT scanning, and should find inspiration for new research objectives; readers who are on the learning curve with applications—here is helpful advice for them; and researchers with greater experience—the field is evolving quickly and it is easy to miss aspects. Our conclusion is that CT scanning of roots and canopies is highly demanding in terms of technology, multidisciplinarity and big-data analysis, to name a few areas of expertise, but eventually, the reward for researchers is directly proportional!

## Introduction

Many discoveries and much research progress have been made in the plant and soil sciences thanks to computed tomography (CT) scanning, from Tollner et al. (1987) and Aylmore (1993) to the 2015 *Frontiers in Plant Science* Research Topic “Branching and Rooting Out with a CT Scanner" (Plant Biophysics and Modeling; http://journal.frontiersin.org/researchtopic/2132/branching-and-rooting-out-with-a-ct-scanner-the-why-the-how-and-the-outcomes-present-and-possibly-fu), with several other pioneering studies and more modern publications in the interval (e.g., Aylmore, 1994; Tollner et al., 1994; Dutilleul et al., 2008; Flavel et al., 2012; Mooney et al., 2012). The timing was thus perfect to compile a summary that highlights the main factors, axes and issues involved in a research field which is multidisciplinary by nature and challenging by necessity.

The promise that CT scanning (originally called “computer-assisted tomography” or “CAT scanning”) was able to see inside soil columns and monitor processes underground in a continuous, non-destructive manner led Aylmore (1994) to announce that the technology, designed for diagnostic purposes in the medical world, “has the potential to resolve the major controversies in soil physics and soil-plant-water relationships.” The accessible challenges mentioned at the time included: understanding soil structure development and water movement in soils and its availability for plant development; documenting root system growth in 3-D space over time; and observing the behavior of soil organisms *in situ.* The application of CT scanning to study the branching pattern as aerial plant structure of interest and its complexity in relation to light interception by the leaf canopy is more recent (Dutilleul et al., 2005).

This perspective on the CT scanning of root systems and leaf canopies is meant to be not technically driven. The importance of a surrounding medium with similar vs. very different density, relative to the plant structure of interest, is discussed first. Then, the distinction between scale of observation and scale of resolution is emphasized because their ratio has implications for (i) the recommended equipment and its settings, and (ii) the results of graphical and quantitative analyses. When a CT scanning dataset has been collected for the root system of a crop plant or the leaf canopy of a small-size tree, the plant structure must be isolated from the surrounding medium (e.g., soil for roots) or be separated from other plant materials (e.g., seed, stem for roots; leaves for branches). Two main approaches and the companion analytical procedures will be described and illustrated with a root system example. Important mathematics and statistics questions [fractal dimension (FD) estimation, analysis of temporal repeated measures] are treated separately. To close our perspective, recent bridging experiments (alias “combo studies”), in which CT scanning plays a key role but is not the only technology applied to plants, are commented upon, under the umbrella of plant phenotyping.

## Root Systems, Leaf Canopies, and X-Ray Doses

In CT scanning technology, material density is essential, as it defines the CT number (CTN) value for a “voxel” (3-D extension of a pixel in 2-D space). With X-ray CT scanning, a CTN is expressed as 1000 times the ratio of the difference between the X-ray linear attenuation coefficients for the voxel and water (numerator) to the difference between the X-ray linear attenuation coefficients for water and air (denominator; the coefficient for air is, in fact, 0); its unit is HU (Hounsfield unit; Kalender, 2000). Positive and negative CTN values represent densities higher and lower than that of water, i.e., the expected amounts of X-rays absorbed are, respectively, greater and smaller than for water. Accordingly, floating plant materials have negative CTN values, between *—*1000 (air) and 0 (water) HU. Furthermore, the air medium surrounding a leaf canopy is lighter than the plant material, whereas the contrary is true for a root system growing in a mineral soil, for which CTN values are much greater than 0 and CTN values of any non-floating plant material (Han et al., 2008, Figure 3). The case of root systems in organic soils is the most challenging because root and soil voxels then have overlapping CTN values (Mooney et al., 2012). Depending on the type of soil, adjusting soil moisture content may be helpful; Lontoc-Roy et al. (2006) thus obtained better results for corn root systems, by CT scanning them in dry homogeneous sand and water-saturated loamy sand in a 2 × 2 factorial design.

Consequently, the amount of X-rays required to penetrate the root system and soil contained in a pot with volume *V* is expected to be greater than the amount required for a leaf canopy with equal volume; “penetration” means that a strictly positive portion of the X-rays emitted by the source is recorded by detectors on the opposite side in the gantry. The question of X-ray dose in plant CT scanning (especially when temporally repeated) has been the subject of a debate between Stuppy et al. (2003) and Dutilleul et al. (2005), and a welcome, informative update was recently provided by Zappala et al. (2013). It is the energy spectrum (also called “tube voltage,” kV) that defines the penetrability of X-rays and their expected relative attenuation while passing through materials; higher-energy X-rays penetrate more effectively but are less sensitive to changing density than lower-energy X-rays, so a compromise must be found (Ketcham and Carlson, 2001). Tube voltage values for medical CT scanners remain in the range of 70–150 kV. On modern industrial CT scanners, settings may allow lower values (60 kV for micro-CT) and values as high as 420 kV. Radiation output increases strongly with tube voltage, but a combination of factors actually represents the radiation level delivered. Those factors also include the tube current (mA), distance from source (cm), total scan time (s), filter nature and thickness (mm). The helical scan option (i.e., when several CT images are constructed from CT scanning data acquired in one rotation) reduces the X-ray exposure time. Using the online software of McGinnis (2002–2009) with 120 kV, 100 mA, 35 cm, 440 s, and a 3-mm Al filter (Régent et al., 2013), we calculated an X-ray dose of 6.86 Gy (6860 R) for the potato seedling of our root system example. According to Zappala et al. (2013), such X-ray dose (<30 Gy) is not damageable for plant growth or soil microbial populations.

## Scale of Observation vs. Scale of Resolution

Effective work in plant CT scanning crucially depends on the scale of observation (different from the statistical concept with same name) and the scale of resolution (Ketcham and Carlson, 2001; Mooney et al., 2012). The size of the object to be CT scanned defines the scale of observation; its volume ranges from m^3^ to mm^3^, with dm^3^ and cm^3^ in-between. Accordingly, the scale of resolution, which is defined by the size of voxels for which CTN data are acquired, ranges from mm^3^ to μm^3^, with (100 μm)^3^ and (10 μm)^3^ in-between. The combination of the two scales defines the type of CT: from conventional to micro-, with high-resolution and ultra-high-resolution in-between.

Interestingly, some scanning systems are said to be micro-CT, but their configuration for the reported experiments provided CT scanning data at the ultra-high resolution at best. The smallest resolution (μm^3^) can be reached with synchrotron X-ray sources, and true microtomography may then allow the detection of hair roots with a diameter of a few μm. That is for a very small part (<1 cm^3^) of the root system, though, and with excessively large CTN datasets to analyze if expanded (Mairhofer et al., 2012). Accordingly, synchrotron-based CT scanning has very originally been established as a technology to assess the filling status of xylem vessels and detect embolisms (Lee and Kim, 2008; Choat et al., 2015), and to unravel anatomical features of the vascular system (Kim et al., 2014). Concerning root systems and leaf canopies, medical CT scanners can provide a finer spatial resolution than anticipated in the X–Y plane of CT scanning (perpendicular to the couch), thanks to a zoom factor option (see Dutilleul et al., 2015, for leaf canopies). Even in this case, it is important that voxels be as cubic as possible, to approach the isotropy condition; the tendency for “isotropic voxels” is being generalized.

**FIGURE 1:**
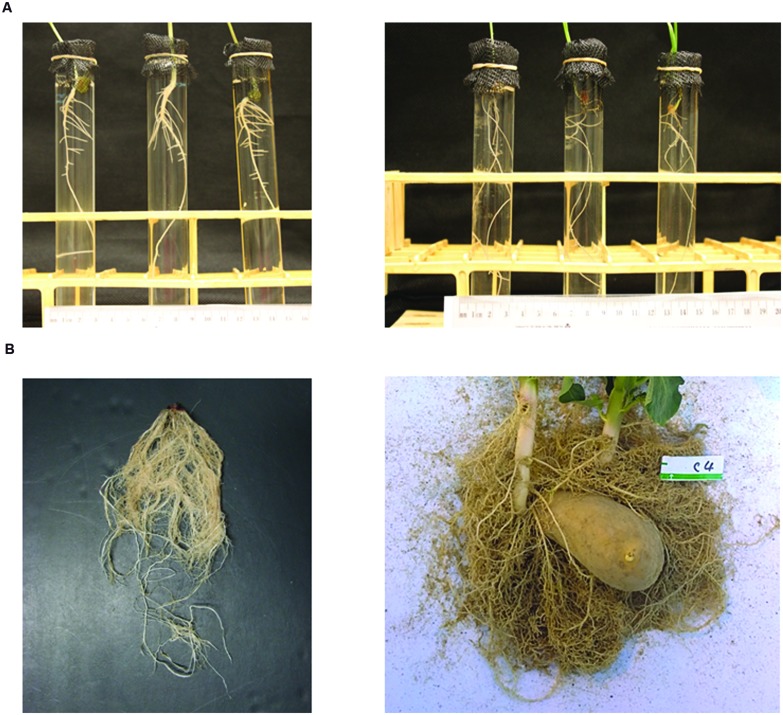
**Photographs of root systems of **(A)** three 8-day-old mung bean *(Vigna radiata)* seedlings (left) and three 3-week-old wheat *(Triticum aestivum)* seedlings (right), grown hydroponically, and **(B)** a 5-week corn seedling (Subramanian et al., 2015) and an 11-week potato seedling (Han et al., 2009), after growing in homogeneous sand in pots, digging and washing, as examples of plant structures to be studied at scales of observation of **(A)** mm to cm and **(B)** cm to dm**.

On medical CT scanning systems, a number of “fields of view” (diameters, cm, in the X–Y plane) pre-define the scale of observation (before application of any zoom factor): e.g., SS (18); S (24); M (32); and L (40). Given a 512 × 512 size of CT image and a zoom factor value (≥1.0), it is easy to calculate the X–Y dimensions of a voxel and see that high resolution is reachable with the 18-cm field of view, for a sufficiently small Z-depth. On the non-medical side, there appears to be a greater flexibility for smaller objects and finer resolution; see, e.g., the ∼2.5-cm scale of observation and ultra-high resolution in Mairhofer et al. (2012, Table 1, first Wheat column). **Figure 1A,B** here shows root systems that would be CT scanned at ultra-high resolution vs. high resolution.

## Analytical Procedures for CT Images and Associated CT Number Datasets

Below, the emphasis is on the isolation of roots from plant-soil CT scanning data and the subsequent construction of 3-D root system images; the cases of hydroponically-grown roots, leaf canopies and branching patterns are easier without being straightforward, the search for interfaces between leaves and branches having its own challenges (Dutilleul et al., 2015). The combination of very fast scans and automated analytical procedures may be the ultimate goal, so that CT scanning technology realizes its potential as a high-throughput technique for the quantification of roots in soils (Flavel et al., 2012), much has been done but there is still much to do regarding analytical procedures. Classically, the root isolation problem in plant CT scanning is presented in a way that allows two antagonist approaches: “top-down”—a first set of root voxels is isolated and neighboring root voxels are joined based on some criterion; “bottom-up”—an initial group of voxels containing candidate root voxels is successively refined to remove all non-root voxels (Mairhofer et al., 2012). That problem can be posed in another way, as we explain hereafter.

**FIGURE 2:**
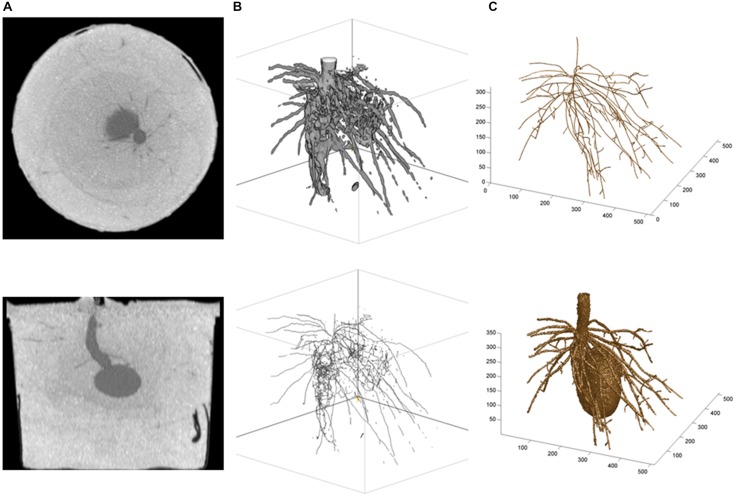
**Illustrations from a potato root CT scanning experiment.** **(A)** Horizontal (top) and vertical (bottom) cross-sectional CT images of the below-ground organs of a potato seedling 4 weeks after seed planting (Han et al., 2009); **(B)** *ImageJ* results: top, Plant volume (i.e., fragments of first-order and secondary roots, and underground part of the stem), obtained by global thresholding applied to the gray tones in CT images—the seed was removed digitally before the beginning of the work in *ImageJ*, and bottom, Skeletal structure obtained with the plug-in *Skeletonize (2D/3D);* and **(C)** *MATLAB* results: top, Skeleton of the root system (i.e., unbroken first-order roots and attached fragments of secondary roots, plus underground part of the stem; thinner roots such as hair roots are missing because they could not be isolated at such scales of observation, dm, and resolution, 100 (μm, which correspond to high-resolution CT scanning; see text, Section “Root Systems, Leaf Canopies, and X-ray Doses”), obtained by tracing roots in CT images with a customized *MATLAB* procedure, and bottom, Plant volume obtained by expansion of the skeleton using the CT numbers of neighboring voxels, with different CTN-threshold values and maximum numbers of layers for first-order and secondary roots (i.e., lower CTN-threshold value and greater maximum number of layers for first-order roots)—the seed was developed from CT scanning data separately and added subsequently. The same 320 (horizontal) raw CT images were involved in the analyses in **(B,C)**; they were used as such for **(C),** and after a preliminary transformation into new image files with 256 gray tones for **(B)**.

Alternatively, two approaches can be developed and followed, depending on whether researchers have access to (i) the CT images only or (ii) the CTN datasets mapped into CT images. Under (i), a graphical and semi-quantitative approach based on the grayscale values in CT images (e.g., 256 tones) is followed, and the open-source package *ImageJ* (The U.S. National Institutes of Health; Rasband, 1997–2014) can be used. Under (ii), a root system skeleton is first traced manually through the CT images (graphical phase) and then, the skeletal roots are expanded in a neighborhood analysis involving the CTNs (quantitative phase); built-in and customized programs written in the technical computing language *MATLAB* (The MathWorks Inc.) have been used for this and other related purposes (Subramanian et al., 2015; see also, e.g., Koebernick et al., 2015; Paya et al., 2015). A potato root system example is presented in **Figure 2**, and we refer to the legend for more technical details. Obviously, the *MATLAB-*type of analysis is less automated than the *ImageJ*-type, even though the expansion from root system skeleton to volume is performed with a customized *MATLAB* program. Accordingly, the results are fragmented *(ImageJ)* vs. continuous *(MATLAB)* in **Figure 2B** (lower panel) vs. 2C (upper panel), and approach (i) can be said “bottom-up” whereas approach (ii) would be “top-down” in classical terms. On the commercial side, the general graphical features of VGStudio Max (Volume Graphics GmbH) were found useful by Flavel et al. (2012), Bao et al. (2014), and Metzner et al. (2015).

At least 100 segmentation methods are documented (see Sezgin and Sankur, 2004; Tuller et al., 2013 in the porous media and soil sciences). They include segmentation by global or local thresholding, when boundaries in grayscale or CTN values are applied to select voxels over a larger or smaller extent. And automated procedures may be aimed to avoid operator bias (Baveye et al., 2010), but are not a substitute to operator intelligence, so semi-automatic procedures may represent an acceptable compromise.

## From CT Scans to Fractals and Repeated Measures Anova

After visualization of the plant materials in the CT scanning data and isolation of the structured part, the resulting 3-D image of the plant structure of interest (root system, branching pattern) provides a basis to estimate its complexity; see **Figures 2B,C** for our root system example. However, to avoid any bias due to the thickness of roots or branches, that 3-D image must be further processed and submitted to a “skeletonization” (reduction of thickness to one voxel). Under the fractal geometry assumption (i.e., within the limits of the spatial resolution of CT scanning data, the structure repeats itself at decreasingly smaller scales; Mandelbrot, 1983), the complexity of the 3-D plant structure can be quantified by its FD, estimated with a cube-counting procedure; the higher (lower) the FD value, the higher (lower) the complexity. To our knowledge, fractal geometry elements were first applied by Lontoc-Roy et al. (2005) for the 3-D analysis of root system images constructed from CT scanning data; comparison to 2-D results obtained from washed roots (destructive sampling) was made in Lontoc-Roy et al. (2006). Recent, detailed plant applications of the cube-counting procedure for FD estimation can be found in Subramanian et al. (2015) for root systems and in Dutilleul et al. (2015) for branching patterns. The distinct information provided by FD, compared with root, leaf and branch traits (e.g., lengths, areas, volumes) that are not structural complexity measures, makes it a key complement to include in the quantitative analysis of plant CT scanning datasets.

If one CT image is the graphical representation of a 512 × 512 matrix of CTN values (∼250,000 entries), then 500 CT images constructed for a root system represent ∼125 million data. From the skeletonized 3-D image of a root system, however, only one estimated FD value should be retained, and from the non-skeletonized 3-D image, only one estimated root volume and one estimated total root length. That means three sample sizes of 1! Although first-order root traits can be measured individually, sample sizes are not as large as they may first seem in plant CT scanning experiments.

Zappala et al. (2013), following Dutilleul et al. (2005), established conditions in which temporally repeated CT scanning is possible with plants. Statistically speaking, temporal repeated measures on the same plant, or “subject” in general terms, do not mean increased sample size. Instead, it implies the use of “subsamples” for each subject. Since these are not random, the statistical analysis of temporal repeated measures requires an adjustment for autocorrelation and heteroscedasticity (heterogeneity of the variance) when the classical ANOVA (analysis of variance) *F*-tests are invalid; they suffer from an inflated rate of rejection of the null hypothesis when true. Modified ANOVA *F*-tests are then performed for within-subject effects (time-related effects in the ANOVA model); classical ANOVA *F*-tests remain valid for between-subject effects such as treatment main effects (Crowder and Hand, 1990; Dutilleul, 2011). When sample sizes are small, a mixed-model analysis is not recommended in general because of asymptotics in the estimation and testing.

## Plant CT Scanning Combined with Other Technologies and Methods of Research

In the 1980s and 1990s, researchers had to adjust to the continuous development of a medical technology, and improve as much as possible the graphical analyses of CT scanning data in non-medical applications; Ketcham and Carlson (2001) provide an excellent review of the efforts made on the problem till then in the geosciences, including corrections to reduce beam-hardening effects in CT images of dense materials. The search for appropriate processing and analysis of CT images in an extended range of fields is fertile ground for computer-science and engineering joint research; see, e.g., the automated graphical procedure involving multiple processing stages, proposed by Entacher et al. (2007) to generate tree-ring profiles from the CT image showing a cross-section of the trunk.

Plant-soil CT scanning is an ideal platform to build bridges between fields, and provide supplementary information to researchers in them. Prior to the advanced applications in phytopathology by Han et al. (2008, 2009), with the common scab-inducing pathogen *Streptomyces scabies* and potato as the experimental crop, a medical CT system was used in soil science by Grose et al. (1996) to measure moisture content in bulk soil and the soil around roots, in order to predict suitable growth conditions for plant pathogenic fungi *Rhizoctonia solani* and *Gaeumannomyces graminis.* Because CT scanning technology is density-based, it cannot resolve solely all the research objectives in some plant studies, and other means or methods of data collection are then required. Thus, the joint use of CT scanning and phenotypic/genetic analyses allowed Bao et al. (2014) to identify a mechanism that plant roots might follow to grow toward available water. Combining shade tolerance indices from the ecological literature with leaf canopy and branching pattern traits measured from the CT scanning data collected for miniature conifers, Dutilleul et al. (2015) found differences in mean values of traits and correlations between traits depending on the leaf type, scalelike or needlelike. In a soil-water-root hydrodynamic study, Koebernick et al. (2015) included root architectures reconstructed from CT scans in a simulation model for water potentials in soil and roots in 3D and water uptake by growing roots at different depths. In a recent phytopathological application, Sturrock et al. (2015) quantified, thanks to CT scanning, the damping-off effects caused by *Rhizoctonia solani* on roots of wheat and oil seed rape, and related their visual assessment of the disease to pathogen DNA quantification in soil using real-time PCR.

Continued advances in CT scanning data collection and CT image analysis algorithms, for root systems under ground level and leaf canopies at interfaces with branches, will make more high-throughput applications and complete plant phenotyping possible, e.g., in greenhouse growing conditions. That is, in a breeding plan, plant structures associated with greater water and nutrient uptake from soil media and higher interception of sunlight will be revealed, but also “after the fact,” new plant varieties resulting from a genomic or biotechnological improvement will see their structures characterized exhaustively. In all of this, a spatio-temporal approach based on careful use of repeated CT scanning is possible, and represents an undeniable advantage. In closing, this is only the beginning of plant CT scanning “combos” and a large number of exciting, bridging experiments may be expected in the next years.

## Conflict of Interest Statement

The authors declare that the research was conducted in the absence of any commercial or financial relationships that could be construed as a potential conflict of interest.
